# Bringing new tools, a regional focus, resource-sensitivity, local engagement and necessary discipline to mental health policy and planning

**DOI:** 10.1186/s12889-020-08948-3

**Published:** 2020-06-05

**Authors:** Jo-An Atkinson, Adam Skinner, Kenny Lawson, Sebastian Rosenberg, Ian B. Hickie

**Affiliations:** 1 Brain and Mind Centre, Faculty of Medicine and Health, The University of Sydney, Sydney, Australia; 2 Computer Simulation and Advanced Research Technologies, Sydney, Australia; 3 Menzies Centre for Health Policy, The University of Sydney, Sydney, Australia; 4 Translational Health Research Institute, Western Sydney University, Penrith, Australia; 5 Hunter Medical Research Institute, Newcastle, Australia; 6 Research School of Population Health, The Australian National University, Canberra, Australia

**Keywords:** Mental health services planning, Suicide prevention, System reform

## Abstract

**Background:**

While reducing the burden of mental and substance use disorders is a global challenge, it is played out locally. Mental disorders have early ages of onset, syndromal complexity and high individual variability in course and response to treatment. As most locally-delivered health systems do not account for this complexity in their design, implementation, scale or evaluation they often result in disappointing impacts.

**Discussion:**

In this viewpoint, we contend that the absence of an appropriate predictive planning framework is one critical reason that countries fail to make substantial progress in mental health outcomes. Addressing this missing infrastructure is vital to guide and coordinate national and regional (local) investments, to ensure limited mental health resources are put to best use, and to strengthen health systems to achieve the mental health targets of the 2015 Sustainable Development Goals.

Most broad national policies over-emphasize provision of single elements of care (e.g. medicines, individual psychological therapies) and assess their population-level impact through static, linear and program logic-based evaluation. More sophisticated decision analytic approaches that can account for complexity have long been successfully used in non-health sectors and are now emerging in mental health research and practice. We argue that utilization of advanced decision support tools such as systems modelling and simulation, is now required to bring a necessary discipline to new national and local investments in transforming mental health systems.

**Conclusion:**

Systems modelling and simulation delivers an interactive decision analytic tool to test mental health reform and service planning scenarios in a safe environment before implementing them in the real world. The approach drives better decision-making and can inform the scale up of effective and contextually relevant strategies to reduce the burden of mental disorder and enhance the mental wealth of nations.

## Background

Mental illness and substance misuse are a significant and growing burden worldwide. The magnitude of the burden has been estimated to be 7·4% of the total global burden of disease in 2010 [1, 2], and there is an alarming worldwide prevalence of mental disorders of 14% in children and adolescents [3]. Mental illness in early life has important implications for the social, family, educational and vocational trajectories of young people and for the ‘mental wealth’ of nations. Mental health influences the degree to which an individual can participate in education, training, the labour market, social relationships, and positive physical health behaviours. At a societal level these factors influence earnings potential, productivity, innovation, economic growth, crime rates, social cohesion, civic engagement, and political stability [4–8]. With the hidden role of mental illness in undermining progress towards achieving sustainable development goals (SDGs) increasingly being recognised, international efforts to improve the mental health of populations, and specifically young people, have intensified [9].

Adoption of the World Health Organisation’s (WHO) Comprehensive Mental Health Action Plan in 2013 [10] and inclusion of mental health in the Sustainable Development Goals (SDGs) in 2015 [11], has given rise to renewed efforts worldwide towards community-based mental health care and strengthening of health systems globally. Specifically, there is an urgent need to close the treatment gap for mental disorders, provide more effective care and improve outcomes in low-to-middle income countries [12–14]. Policies and plans have focussed on moving away from centralised and specialised clinics toward integration of mental health care with the general healthcare system, and improving funding models, governance, system design and accountability [12, 14–20]. However, best approaches for achieving this in resource-limited settings are unclear. ‘Developed’ countries are still challenged by a high burden of mental disorders and struggle to articulate best approaches to system design, system strengthening and resourcing allocation. They struggle to deliver improved access to the quality mental health services required to achieve real and lasting impacts on quality of life, life expectancy and social and economic participation.

At the individual level mental disorders are complex. For health system responses to be effective they must account for this complexity through provision of timely, coordinated and patient centred care. Recently, attention has been called to the failing acute-focussed, increasingly specialised, ‘diagnosis-evidence-based-practice symptom-reduction’ paradigm that dominates mental health service delivery [21]. This paradigm is argued to be too expensive to be sufficiently scaled to meet the significant proportion of the population with mental health needs. Critics have also questioned the degree to which a focus on diagnosis and symptom reduction will achieve the outcomes relevant to those living with mental disorder; such as, improved educational, vocational, social, and cultural participation [21]. New platforms are being developed that recognise the spectrum and dynamics of mental illness, and are helping to address both timely assessment, diagnosis and coordinated clinical care, as well as supporting broader functional needs of individuals that facilitate their management of a mental disorder (such as securing appropriate housing or supported employment). They provide consumers with appropriate care for a particular stage of illness, thereby reducing the risk of escalation in severity [22, 23]. Such platforms aim to increase access to standardized, broad-based assessment, and identify and track changing consumer needs over time (including clinical, social, educational and vocational requirements). These needs are then matched to personalized care options without having to wait for an appointment, enhancing the quality of the care provided to consumers and coordinating professional interactions with clients [24]. Australian multi-site, regionally-based trials to evaluate the value and effectiveness of online and digital tools to support coordinated clinical service delivery and personalised care are showing early promise [24]. However, the optimal effectiveness of such platforms is dependent on the timely availability and careful balance of community-based programs and services with tertiary mental health services as well as adequate regional infrastructure and an appropriately trained and supported workforce. This is a balance that regional health systems find challenging to establish and maintain given the complexity of the decision-making environment.

In this viewpoint, we suggest a key to unlocking this problem lies in more routine engagement with advanced decision support tools. These tools, using dynamic systems modelling and simulation, can facilitate informed strategic investment decisions, capitalising on limited mental health resources while accounting for changing community needs over time. We argue that such tools are best customised, governed and applied regionally, while drawing on standardised, scalable components, thus helping to solve the common challenges faced in both developed and developing nations, as well as the major variations we faced within nations. These approaches can help to realise the full potential of future investments in mental health in improving clinical and functional outcomes across populations.

## Discussion

### Challenges of achieving impact: the Australian experience

Mental illness in Australia is the largest single cause of disability, with as many as one in five people aged 16 to 85 years experiencing a mental illness in any 1 year [25]. Productivity losses from those living and working with mental illness may be as high as $14 billion per annum [26]. There is a non-linear relationship between the level of an individual’s psychological distress and productivity. Low levels of distress have been estimated to result in productivity falls of 6·4%, and as distress increases to moderate, and then to high levels, the productivity falls are 9·4% and 20·9% respectively [27]. Suicide results in $1·7bn of lost lifetime productivity [28]. There is a clear social, economic, and moral imperative to effectively improve national mental health.

Each year in Australia over $9·0 billion is spent on mental health related services, with approximately 60% funded by state and territory governments, 35% by the Australian Government, and about 5% by private health insurance funds [29]. This does not include broader mental health-related costs, such as the Disability Support Pension and Carer Payment and allowances, nor funding for services provided by non-government organisations, philanthropic investments or out of pocket costs paid by patients themselves [29]. In addition, over the last decade, hundreds of millions of dollars have been spent on mental health system reforms to make services and preventive interventions more effective, efficient, and culturally appropriate. Significant government and philanthropic investments are also being made in research to identify the most effective strategies [30].

Despite recognition of the potential benefits of improved mental health, and despite decades of reforms and investments of this scale, rates of mental illness in Australia are not decreasing [31]. There is little agreement regarding the reasons for this reality or the appropriate strategies now required to address the complex, persistent problem of mental illness in this country [32]. A range of perspectives have been offered in academic discourse, in the media, and in the findings of successive statutory inquiries [33–35] which include:

- That policy rhetoric has not been supported by planned and funded implementation of reform;
- That there is insufficient overall funding;
- That funding is inappropriately distributed across acute care in public hospitals, primary care, and community-managed mental health needs;
- That services are poorly distributed across geographic and socioeconomic strata;
- That there are insufficient investments in workforce, appropriate training, and infrastructure such as psychiatric beds;
- That interventions are poorly targeted across the lifespan;
- That investments are being made in programs and services that lack evidence, with a push to focus only on those that are evidence-based;
- That services demonstrated to be effective have failed to be delivered at scale;
- The lack of impact has been attributed to the failing acute-focussed, increasingly specialised, ‘diagnosis-evidence-based-practice symptom-reduction’ paradigm that dominates mental health service delivery in this country and elsewhere [21]. It is argued that investments should be made in a variety of cross-sectoral models of supported education, employment and personalised care that are focussed on achieving functional improvements in addition to diagnosis and symptom reduction [21]; and
- A lack of strong accountability which has long been an integral element of mental health action plans [36].

Calls for further reforms from experts and stakeholders holding this array of perspectives presents significant challenges for governments in determining what should be done, and how best to allocate current and future mental health investments. The task of mental health reform is further challenged by the division of roles and responsibilities between Federal, State and Territory governments, regional primary health networks, and private and non-government sectors, creating a level of system complexity that makes the provision of integrated and coordinated, client-centred services and interventions, and their evaluation difficult [29]. This complexity is further amplified when making the close association between mental health and the social determinants of health. Without tools to make sense of this complexity, decision making in mental health has often relied on an inadequate mixture of historical precedent, best guess and trial and error.

Taking up the recommendations of the 2014 review by the National Mental Health Commission [37], the Federal Government called for appropriate focus on regional planning, commissioning and implementation of mental health and suicide prevention programs and services, and utilisation of new technologies, research, and systematic national evaluation [35]. On 1 July 2015, the Australian Government established 31 Primary Health Networks (PHNs); independent not-for-profit primary health care organisations located across Australia. PHNs support the primary care system (including GPs, nurses and allied health practitioners to improve patient care) as well as improve coordination between different parts of the health system, such as between hospitals and community-based care providers. The role of PHNs is to commission, rather than provide programs and services, but they work closely with providers to monitor performance, implement change and improve the coordination of care to ensure patients receive ‘the right care, in the right place, at the right time [38].’

However, national decision makers still face serious challenges in selecting from multiple system reform options and decision makers at a regional level now also face real challenges in making appropriate commissioning decisions to deliver effective mental health programs and services to meet local needs. These challenges include the complexity of mental illness and its multisectoral determinants, workforce capacity restrictions, numerous options for investing programs and services, geographical variation and changing population needs over time, competing views and agendas about what works locally and what should be done, and the timeliness of data. In mental health and across public health more broadly, these complex challenges have resulted in a move towards the implementation of broad strategies, based on the rationale that if more evidence-based programs and services are implemented, then the impact is likely to be greater [30, 39–42]. However, such strategies often fail to be cognisant of the delicate balance and interaction of core elements of the mental health system in a particular context, and the impact that programs and services acting on one part of the system can have elsewhere in the system. Broad strategies can lack focus, result in service systems that are crowded and difficult to navigate, or lack sufficient actual investment in time, resources and capacity to implement in specific geographic and socio-economic contexts. Consequently, this approach may actually undermine the potential impact of investments by spreading available resources too broadly over a range of poorly targeted and coordinated programs and services [30].

We contend that the absence of an appropriate predictive planning framework is one critical reason Australia has failed to make substantial progress in mental health reform over the past two decades. Addressing this missing infrastructure is vital to Australia’s future national capacity to intelligently guide and coordinate national and regional (local) investments and ensure that mental health resources are put to best use. Moreover, the absence of predictive planning frameworks in resource-limited settings represents a deficiency in the strategic capability required to understand best approaches to national system design, and local system strengthening and resourcing allocation; undermining the ability to achieve mental health SDGs.

### Limitations of traditional analytic tools to support decision making

Traditional analytic tools for prioritising programs and services and their targets have important limitations when applied to complex problems such as determining how best to reduce mental disorder and suicidal behaviour in the population [43]. First, current methods determine the comparative burden each risk factor contributes in a given population, and the proportion of that condition that could be averted by targeting high-burden risk factors [44]. The assumptions underpinning these estimates are that risk factors are independent, and relationships between risk factors and outcomes are unidirectional, linear, and constant through time [43]. However, complex problems are characterised by interaction of risk factors, feedback loops (for example, unemployment contributes to depression and depression can prevent gainful employment), thresholds (or breaking points), and changing behaviour over time, all of which violate the assumptions of traditional analytic methods [45]. Second, traditional decision analytic tools which seek to prioritise programs and services on the basis of their comparative costs, benefits, or return on investment, do not adequately account for population dynamics, behavioural dynamics, service or workforce dynamics, the variation in their impacts over time or the non-additive effects of combining them [46]. These limitations make them ill-suited for informing decision making to address complex public health problems. As a result the application of traditional methods can lead to unrealistic expectations of the potential impact of evidence-based interventions in real-world settings [43].

### Lessons from non-health sectors to guide mental health system reform, investment decisions and service planning?

It is common for sectors outside of health, such as engineering, defence, economics, ecology and business, to use dynamic systems modelling and simulation (computer simulation) prior to making significant investments or reforms. These models forecast the likely impact of investments and determine the viability and comparative effectiveness of alternative strategies before implementing them in the real world. They simulate and help solve complex strategic and operational problems, optimise system design and resource management, and improve efficiency and public safety [47]. Additionally, systems modelling and simulation has been instrumental in other sectors in contributing to scientific and industrial advances. Complex technological exploits such as space and planetary exploration could not have been achieved without the use of computer simulation [48], and it is difficult to estimate how many lives have been saved globally by our ability to model, simulate and forecast the path, severity and duration of significant weather events [49]. Computer simulation in these key areas evolved over many decades to achieve increasing levels of forecast accuracy and utility for decision makers. Successful evolution of these sectors - despite, or because of, complexity - occurred as a result of a willingness to embrace a model-learn-adapt cycle [50]. In business, systems modelling and simulation is used to better understand the costs of maintaining the status quo, the costs of reactive rather than proactive strategies, the structural impediments to innovation and performance, and the unintended consequences that can arise from ‘rational’ solutions [51].

Dynamic systems modelling and simulation has supported the control and elimination of global infectious diseases, contributed to epidemic and bioterrorism preparedness [52–55], and aided decision making to reduce non-communicable disease [56] and humanitarian operations [57]. However, for the most part health and social sectors, and the public service in general, have lagged behind in the routine use of these approaches to support policy, planning, monitoring, and evaluation [56]. As in other sectors, the application of systems modelling and simulation can drive better decision-making in mental health and suicide prevention by facilitating the exploration of the likely impact of alternative system design and service planning scenarios before they are implemented in the real world. Recent applications [47, 56, 58–67] of these advanced decision support tools have generated new knowledge and insights that are only possible when we use systems thinking and systems modelling methods to bring together the different pieces of a complex puzzle. This puzzle has many pieces, including, for example, research into the broader social and economic determinants of mental health and suicidal behaviour, service barriers and facilitators, and assessment of local needs, evidence regarding effectiveness of mental health models of care and population-based programs, together with disparate, multi-agency data sources, expert and local knowledge, and the deep understanding and unique perspectives of those with lived experience. Insights from emerging mental health systems modelling applications in Australia have included:

- the identification of leverage points (areas in the system where targeted interventions deliver greater than anticipated effects);
- an understanding of potential unintended consequences of programs and services; sources of system inertia and delay that can limit the population impact of ‘effective’ evidence-based interventions;
- dynamic interrelationships between tertiary and community-based service capacity that reveal important threshold effects for suicidal behaviour;
- the interaction of programs and services producing synergistic or non-additive effects; and
- the optimal combination, targeting (e.g. high-risk groups or events vs. whole-of-population), timing, scale, frequency and intensity of investments in screening, treatment, mental health promotion strategies, and/or reducing the broader drivers of psychological distress and suicidal behaviour including substance abuse, unemployment, domestic violence, homelessness, childhood trauma etc.

For example, in 2017 a system dynamics model was developed in partnership with Western Sydney Primary Health Network and their stakeholders to inform decision making for local investment in suicide prevention programs and mental health service planning [59, 63]. This model simulated cuts to psychiatric beds under different conditions related to community-based service capacity, forecasting the likely impact on suicide rates over the next 10 years [60]. Findings suggested that not all reductions to beds result in increases in suicide, and that a dynamic ‘tipping point’ exists that is influenced strongly by the availability of community-based mental health services [60].

**Fig. 1:**
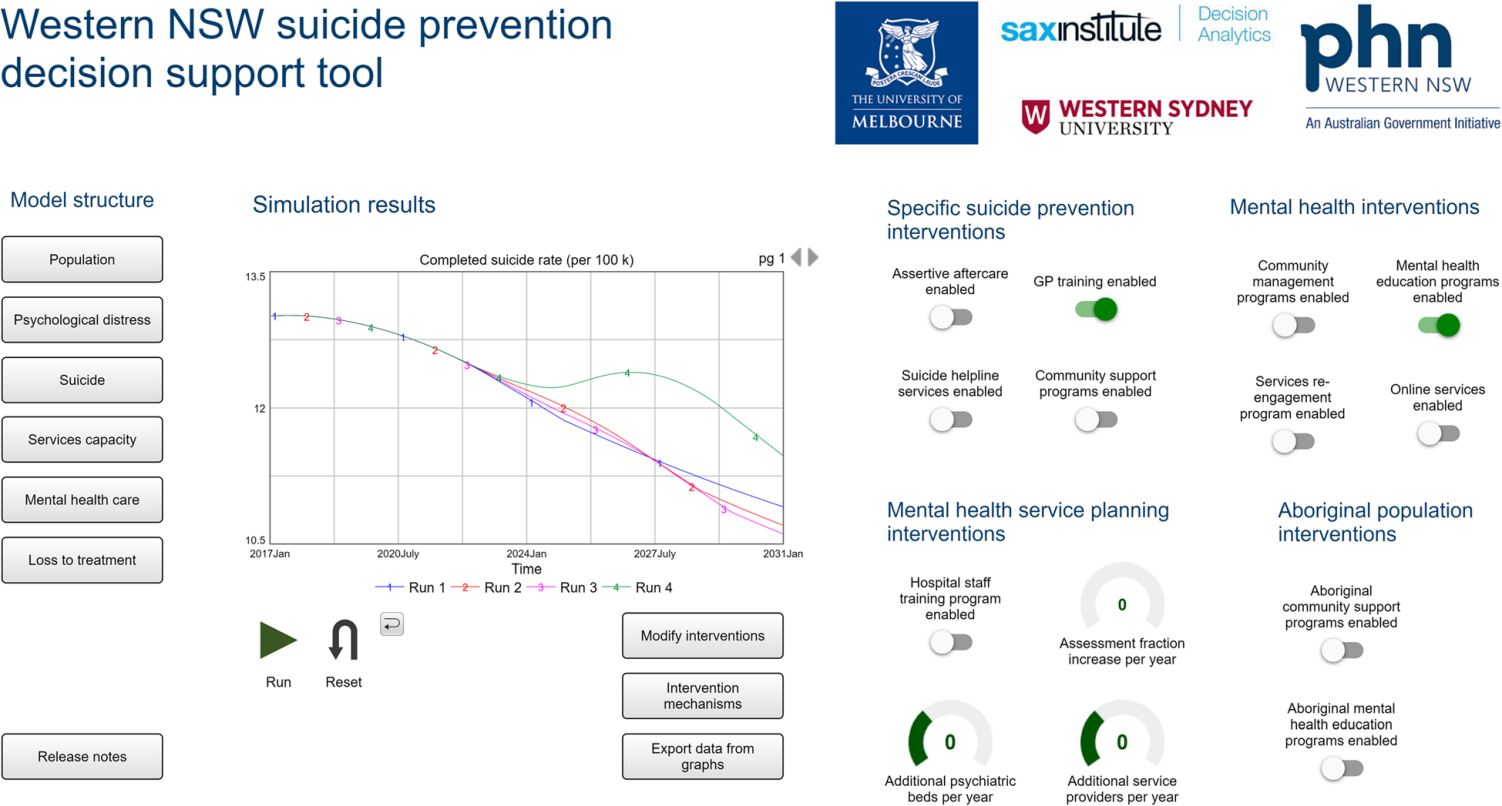
The dynamic systems model of suicidal behaviour in the rural population catchment of Western NSW Primary Health Network. (1) Blue line = baseline (business as usual scenario); (2) red line = GP training; (3) pink line = mental health education programs; (4) green line = GP training plus mental health education programs

Another model was developed for the rural population catchment of Western New South Wales (unpublished). This demonstrated the unintended consequences of implementing general practitioner training (to recognise signs of suicide ideation and refer to appropriate services) together with mental health education programs (aimed at improving mental health literacy and help seeking). Figure 1 shows the unexpected increase in suicide deaths forecast to occur in implementing these two interventions together. This is explained by a lack of service capacity to meet the increase in service demand generated. This example highlights the importance of context. Effective planning depends on understanding the critical balance and timing of implementing combinations of new programs and services, alongside the strengthening of existing service capacity a given local context. Understanding such dynamics and the quantitative impacts of alternative implementation scenarios are exceedingly difficult with the application of static, linear analytic approaches alone.

The task of transforming mental health systems and scaling up effective and contextually relevant strategies is common across most countries. There has never been a more important time to leverage the sophisticated decision analytic tools that are emerging in mental health research, and use them to test strategies in the safety of a virtual environment before exposing populations to solutions whose likely impacts are uncertain; ‘experience is an expensive school … ’.

### The importance of a participatory approach (co-design)

Participatory co-development of these sophisticated decision support tools has been an important component of the collaborative work of the authors, engaging multidisciplinary, multisectoral stakeholders from academia, policy planning, clinical practice, economics, the private and community sectors and people with lived experience. This broad representation of system actors and the practice of encouraging stakeholders to consider potential unintended consequences and inadvertent introduction of perverse incentives, are important for keeping in sight impacts of mental health initiatives on the wider health and social systems. The participatory process has facilitated communication and intellectual exchange, advanced contentious debates, built consensus among stakeholders, aided transparency and model credibility. It has also driven the translation of model outcomes to wider audiences to garner broader support for collaborative action for implementation. The interactive interfaces of the models (Fig. 1) allow stakeholders to run forecasts and collectively weigh up the trade-offs of alternative intervention combinations by exploring their relative impact on a range of population-level mental health, educational and vocational outcomes. Disparities between population subgroups (such as on the basis of indigenous status, socioeconomic status, or age groups), service use and health system burden, cost-benefit estimates, and productivity gains can also be explored. In bringing together researchers with the end users, and deeply engaging them in the process of developing these sophisticated but realistic decision support tools, our approach has knowledge mobilisation as a guiding principle [68–70] with direct policy and planning impacts.

### Embedding systems modelling and simulation in the policy / planning cycle

Systems modelling and simulation is applicable across developed and developing country contexts with software platforms that are compatible with standard laptops or desktops, and interfaces that are increasingly making the structure, logic and assumptions of models understandable by lay audiences. Modellers work in multidisciplinary teams to ensure that the final tools are robust, customised to capture regional demographics, service structure and dynamics, and are fit-for-purpose. Once developed, dynamic systems models can be used as an interactive ‘what-if’ tool to test the likely impacts of alternative reform, investment and program and service options over the short and long term, and can be retained as an ongoing decision support asset [46].

These tools can help regional and national decision makers determine where, when, and how best to target and allocate investments, and with what intensity, for a given context. After deployment, systematic monitoring and evaluation can then determine the extent to which the modelling corresponds with real-world outcomes over time and how intervention strategies compare with forecast outcome targets. Information from monitoring and evaluation is used to refine model parameters (*data assimilation*) to improve its forecast capabilities and guide subsequent decision-making in a timely and proactive way (providing a continuous improvement framework). Dynamic systems modelling and simulation embedded in monitoring and evaluation cycles provide the necessary decision analytic infrastructure to guide sustained investments, strengthen local mental health and suicide prevention systems, and reduce the fragmentation of mental health programs and services.

## Conclusion

National and local planners urgently need better decision support tools and new skills if they are to drive positive change. Currently, much mental health research that informs decision making emphasises single elements of care, individual treatment programs and static, linear, program logic-based evaluation approaches. These approaches, which assume a simple additive effect of interventions, are being used to derive spurious estimates of likely impacts of both regional and national programs. They fail to properly reflect local dynamic context and are inadequate for supporting judicious system design and strengthening and investments or allocation and management of limited mental health resources.

This view is consistent with that expressed by the 2014 US National Action Alliance for Suicide Prevention Research Prioritization Taskforce [71], which concluded that a genuine evidence-based research agenda needs to utilise *prior* modelling to demonstrate how specific activities will contribute impact *at scale*. The deployment of scalable dynamic systems models will bring a necessary and overdue discipline to national and regional investments in mental health in high- and low-to-middle income country contexts, delivering better outcomes for individuals and communities. It will provide a blueprint for next generation investments in mental health and contribute to unlocking the ‘mental wealth’ of nations.

## Data Availability

Not applicable.

## Abbreviations

SDG: Sustainable Development Goal
WHO: World Health Organisation
GP: General Practitioner
PHN: Primary Health Network
